# Changes in first entry to out-of-home care from 1992 to 2012 among children in England☆

**DOI:** 10.1016/j.chiabu.2015.10.020

**Published:** 2015-11-14

**Authors:** Louise Mc Grath-Lone, Lorraine Dearden, Bilal Nasim, Katie Harron, Ruth Gilbert

**Affiliations:** a Administrative Data Research Centre for England, University College London, London, UK; b Institute of Education, University College London, London, UK; c Department of Health Services Research and Policy, London School of Hygiene & Tropical Medicine, London, UK; d UCL Institute of Child Health, University College London, London, UK

**Keywords:** Looked after children, Out-of-home care, Foster care

## Abstract

Placement in out-of-home care (OHC) indicates serious childhood adversity and is associated with multiple adverse outcomes. Each year 0.5% of children in England live in OHC but evidence is lacking on the cumulative proportion who enter during childhood and how this varies over time. We measured the proportion of children born between 1992 and 2011 who entered OHC, including variation in rates of entry over time, and explored the determinants of these changes using decomposition methods. We also described changes in placement type, duration and stability. By age 18, 3.3% of children born 1992–94 entered OHC. This proportion varied by ethnicity (1.6% of White vs. 4.5% of Black children born 2001–03 entered OHC by age 9, 95% CI [1.5–1.7] and [4.4–4.6], *p* < 0.001) and increased over time (0.8% of children born 2009–11 entered OHC by age 1 vs. 0.5% born 1992–94, 95% CI [0.7–0.9] and [0.4–0.6], *p* < 0.001). This overall increase was driven primarily by the increased rate of entry among White children and not by concurrent changes in the population’s ethnic composition. The proportion of children entering OHC in England is increasing and characteristics of the care they receive are changing with earlier intervention and longer, more stable placements. Further research is required to understand the reasons for these changes in practice and whether they are cost-effective, sustainable, and improve outcomes for children and society.

In England, a looked after child is one who is in under the care of a local authority. Under the Children Act 1989, children who are at risk of or are experiencing significant harm (e.g., due to neglect or abuse) can be voluntarily accommodated or compulsorily removed from their parents by a court order. Approximately 60,000 children in England are looked after in OHC each year (Department for Education, 2015) representing 0.5% of the child population. Recently Ubbesen, Gilbert, and Thoburn (2015) reported that 1.6% of children in England born 1992–94 entered OHC by age 16. However, this study did not include children who were placed in care voluntarily and the use of OHC has increased over time, particularly among infants (Gilbert et al., 2012). Therefore the cumulative proportion is likely to be higher; for example, 3.6% of children born in 1970 spent time in OHC during their childhood (Viner & Taylor, 2005). Ethnic disproportionalities in the use of OHC have been described in other high-income settings, such as the United States (Magruder & Shaw, 2008), Canada (Fallon et al., 2013), and Australia (Tilbury, 2009), with indigenous aboriginal populations and ethnic minorities significantly over-represented. Despite recent increases in the proportion of non-White ethnic minorities in the population (Centre on the Dynamics of Ethnicity, 2012), such variation has not been well-explored in England.

Placement in out-of-home care (OHC) indicates serious childhood adversity sufficient for the State to assume responsibility for a child. Children entering OHC are a vulnerable group and most continue to experience problems whilst in care and after they exit, though the reasons for this are complex. Thus, being in OHC is associated with multiple adverse outcomes both in childhood and in later life (Akister, Owens, & Goodyer, 2010; Richardson & Lelliott, 2003) including poorer health (Ford, Vostanis, Meltzer, & Goodman, 2007; Goodman, Ford, Corbin, & Meltzer, 2004; Martin, Ford, Goodman, Meltzer, & Logan, 2014; Meltzer, Gatward, Corbin, Goodman, & Ford, 2002), risky behaviour such as smoking, drinking, and drug taking (Meltzer et al., 2002), and higher rates of teenage pregnancy and premature death (Vinnerljung & Sallnäs, 2008). Children in OHC also have lower educational attainment (Botchway, Quigley, & Gray, 2014; Vinnerljung & Sallnäs, 2008), are more likely to be permanently excluded from school (Viner & Taylor, 2005), and are less likely to have good relationships with their peers (Meltzer et al., 2002). As adults, they are more likely than their peers to be unemployed (Viner & Taylor, 2005), imprisoned, homeless, or a victim of violent crime (Pritchard & Butler, 2000). Age at entry to OHC, placement setting, and stability of care are associated with better or worse outcomes (Tarren-Sweeney, 2008). For example, children in family foster care have better mental health outcomes than those in residential group care (Meltzer et al., 2002) while multiple placement changes are associated with a higher prevalence of psychiatric disorders (Ford et al., 2007).

To plan future services and allocate increasingly scarce resources, it is important to understand what proportion of children are placed in OHC and what drives changes over time in rates of entry to care. Exploring the type of care provided to children is also important as this affects a wide range of health, social, and educational outcomes, as well as the financial costs of care provision. We aimed to use national administrative data to describe how many children in England enter OHC by age 18 including variation by broad ethnic group. We also sought to investigate the determinants of changes in the proportion of children entering care over time vis-à-vis concurrent changes in the population’s ethnic composition, and to describe variation in the characteristics of care known to influence outcomes, namely placement type, duration, and stability.

## Method

### Data Sources

Data related to children in care in England has been collected routinely by the Department for Education since 1992. However, complete care histories are only available for those with a day of birth divisible by three due to data collection restrictions between 1998 and 2003. The dataset used in this study described all episodes of care between January 1, 1992, and December 31, 2012, (including placement type, legal status, reason child was looked after, gender, date of birth, and ethnicity) for 92,190 children born between January 1, 1992, and December 31, 2011. This sample represented one-third of all looked after children. Children who were looked after but remained with their parents or whose placement location was unknown were excluded from analyses, as well as children in OHC for respite reasons (as they typically have complex health needs). To calculate rates of entry to OHC, denominator data by gender, ethnicity, and single year of age was derived from mid-year population estimates. As mid-year estimates were not available by ethnicity in later years (Office for National Statistics, 2015), ETHPOP data (Wohland et al., 2015) was used as an alternative source of denominator data from 2001.

### Cumulative Proportion of Children Entering Out-of-Home Care

First entry to OHC was defined as the start date of a child’s first episode of OHC for non-respite reasons. If a period of respite care was concurrent with OHC for non-respite reasons (i.e. it had been used to ease a child into OHC for other reasons such as neglect) the start date of the respite episode was considered the first entry to OHC. Entry to OHC was analysed by age and grouped year of birth (e.g., 1992–94 to 2009–11). The numbers entering care at each age from infants (<1 year) to 17 years were counted and multiplied by 3.05 to adjust for the one-third sample. To calculate the age-, gender-, and ethnicity-specific cumulative proportions, the number of children who had entered OHC was divided by the average number of children who would be that age in the relevant calendar year. For example when calculating the cumulative proportion of children born in 2000 who had entered OHC by age three, the denominator was the average of the number of infants born in 2000, 1 year olds in 2001, 2 year olds in 2002, and 3 year olds in 2003. This approach accounted for entry and exit of children from the denominator over time due to immigration, emigration and death. Rates of entry by ethnicity were not calculated for children born before 2001 due to the high proportion with unknown ethnicity (18.4%).

The composition of the United Kingdom’s population changed dramatically during the study period with a doubling of non-White ethnic minorities from 7% in 1991 to 14% in 2011 (Centre on the Dynamics of Ethnicity, 2012). Therefore, we hypothesised that any overall increase in the proportion of children entering care may be an artefact of the changing ethnic composition of the child population, and attributable to an increase in the number of black and mixed ethnicity children who are more likely to become looked after. To explore this possibility, variance in the cumulative proportion of children entering OHC over time was decomposed (Gibbons, Overman, & Pelkonen, 2014) into changes in (a) the ethnic-specific rates of entry to OHC and (b) the ethnic composition of the child population (see Supplementary Box 1 for further details).

### Characteristics of Out-of-Home Care

Type, duration, and stability of OHC placements are difficult to describe as multiple entries and exits over childhood are possible. Placement at first entry was used to describe variation in characteristics of care by age and over time. Characteristics of care were explored for the two years following first entry to care as we felt this timeframe was sufficiently long to explore stability and duration of long-term placements but also allowed more recent changes in policies and practices to be explored. For these analyses, children born in three years across the period for which data was available (1992, 2000 and 2008) were selected (*N* = 13,700). Placement setting at first entry to OHC was grouped into four broad categories: (a) family care: placed for adoption and kin or stranger foster care; (b) group care: children’s home, heath-related residential accommodation, residential school, and other supported accommodation; (c) independent living, and (d) other (including in custody). As children may be temporarily placed in one care setting while waiting for a more appropriate setting to become available, if a child’s placement changed within seven days of first entry the subsequent placement was used for this analysis. Children who entered care for the first time after December 31, 2010 or after their 16th birthday were excluded as they could not have two years of follow-up. Variation in type of placement and proportion of children with more than one placement change by year of birth groups and age group was evaluated using chi^2^ tests and variation in duration of care evaluated using ANOVA. All analyses were carried out using Stata v13.

## Results

### Sample Characteristics

The study sample comprised 84,674 children placed in OHC for non-respite reasons (see Supplementary Fig. 1). Most children entered OHC for the first time through voluntary placement under Section 20 of Children Act 1989 (see Table 1); however, this proportion decreased over time (e.g., from 73.0% of infants born in 1992–94 to 53.7% of those born 2009–11, *p* < 0.001). The majority of children became looked after for reasons related to abuse or neglect except adolescents aged 16 or older who were most likely to enter due to absent parenting.

### Cumulative Proportion of Children Entering Out-of-Home Care

**Fig. 1:**
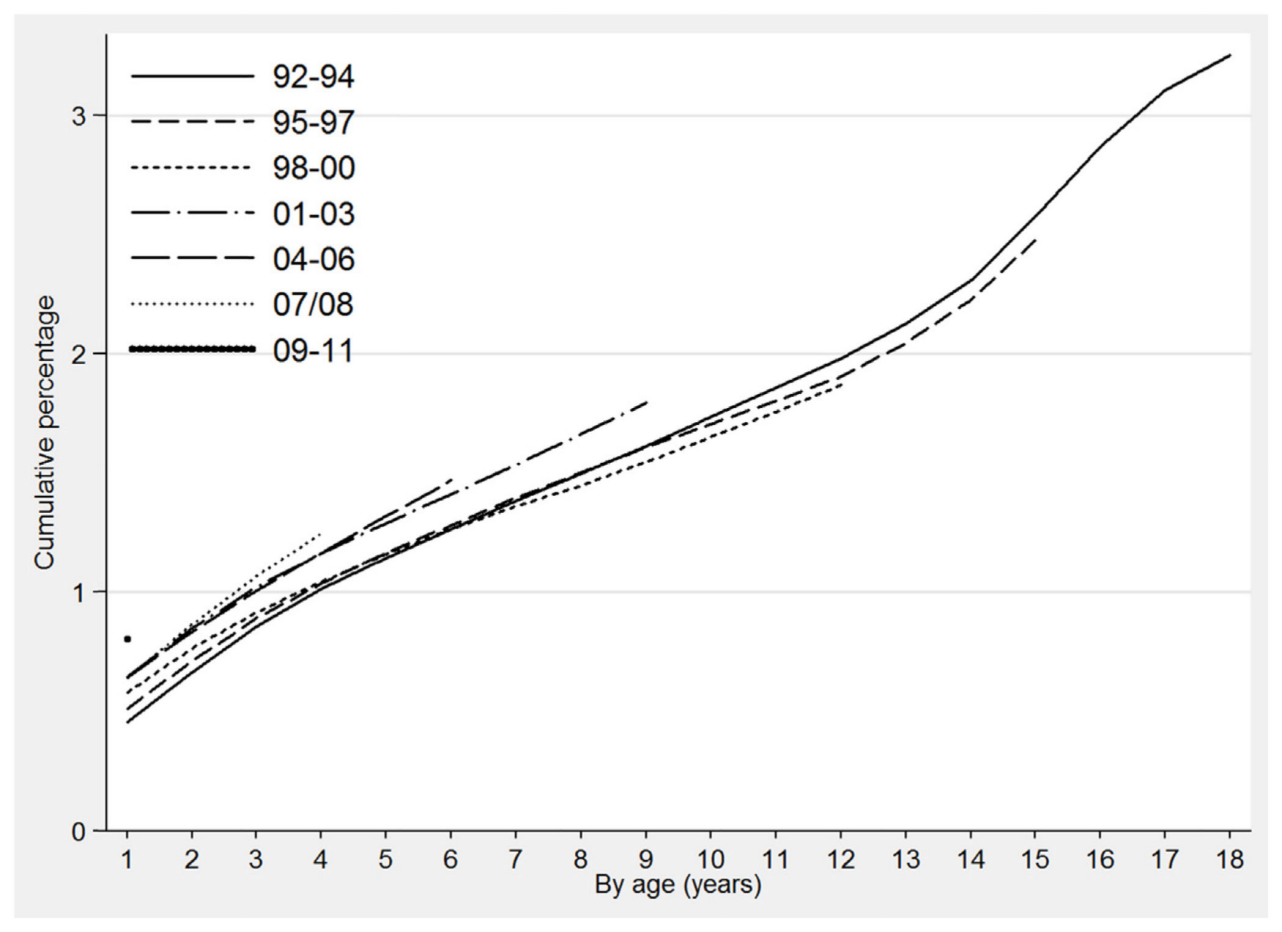
Cumulative proportion of children entering out-of-home care by grouped year of birth and age.

By age 18, 3.3% of children born in 1992–1994 had entered OHC (see Fig. 1). Some gender variation was evident with 3.5% of boys becoming looked after during their childhood compared to 3.0% of girls (*p* < 0.001, see Table 2). The proportion of children entering OHC also varied significantly by ethnicity (see Table 2). Among children born in 2001–03 (the earliest year of birth group for which ethnicity was recorded for >99% of children), rates of entry by age nine were lowest in White (1.6%) and Asian (0.8%) children compared with Mixed (4.2%) and Black (4.5%) children (*p* < 0.001). Over time the proportion of children entering OHC increased significantly; 0.8% of children born in 2009–11 entered care by age one compared with 0.5% of those born in 1992–94 (*p* < 0.001). As the proportion of non-White ethnic minorities in England doubled during the study time period we hypothesised that the overall increase in the proportion of children entering care may be an artefact of the changing ethnic composition of the child population, and attributable to an increase in the number of Black and Mixed ethnicity children who are more likely to enter care. However, when the overall increase was decomposed into components attributable to changes in (a) population weights and (b) ethnic-specific rates of entry to care, the increase in the proportion of children who were non-White was found to have had a negligible effect. Instead the increase over time in the rate of entry to care among White children was the main determinant of the overall increase in the proportion of children in OHC (see Table 3). Among infants, the increase in the rate of entry among White children between 2001–03 and 2009–11 accounted for an absolute increase of 0.15 percentage points in the overall cumulative proportion entering OHC, while changes in other ethnic-specific rates or in the ethnic distribution of the population accounted for changes of less than (+/−) 0.03 percentage points. The increase over time in the proportion of children entering OHC appears to be due to a small yet significant increase in the rate of entry to OHC among white children and not the changing ethnic distribution of children in England.

### Characteristics of Out-of-Home Care

A small number of children (2.8%) changed placement type within one week of first entry to OHC and the majority subsequently moved to stranger or kin foster care settings. Type of placement varied with age at first OHC entry (see Table 4(i)). The majority of infants were placed in a family care setting (i.e. foster care or placed for adoption): almost 10% were placed in a group care setting, primarily health-related residential care settings including mother and baby units. Compared with infants, 1–4 year olds were more likely to be placed in family care settings and the proportion placed in group care settings (e.g., children’s homes, residential schools, etc.) increased with age. Over time, young children (1–10 year olds) were increasingly placed in foster care rather than group care settings. More than a third of children entering OHC for the first time aged 16 or older were placed in independent living.

The number of placement changes also varied by age at first entry (see Table 4(ii)) and adolescents were most likely to experience more than one placement change in the two years following their first entry to OHC. Stability of placements improved over time for older children; for example, 38.4% of 1–4 years olds born in 1992 had more than one placement change compared to 13.9% of those born in 2008 (*p* < 0.001). However, no improvements were over time were evident among infants with almost one in three experiencing multiple placements in the two years following first entry to OHC.

The number of weeks spent in care during the two years following first entry to OHC varied by age group with children who entered for the first time aged 5–10 years spending the longest time (see Table 4(iii)). Over time, the average duration of care in the two years following first entry to OHC increased for all children; for example, the mean number of weeks in care increased from 49 for infants born in 1992 to 70 for those born in 2008.

## Discussion

Our study is the first to describe the cumulative proportion of children entering OHC in England. By age 18, 3.3% of children in England experienced at least one placement in OHC and significantly higher rates of entry were evident amongst children of Mixed or Black ethnicity. From 1992 to 2012, the proportion of children entering OHC increased, driven primarily by a small but significant change in the rate of entry to care among White children. There were also changes over time in the characteristics of care. Children were increasingly placed in family type settings and first placements became longer and more stable.

An important strength of this study is that issues of recall or selection bias associated with survey-based studies of entry to OHC were negated through the use of national administrative data that included all looked after children in England and did not rely on self-report by carers or care leavers. Furthermore, the longitudinal nature of the dataset allowed changes over time in rates of entry to care and its characteristics to be reliably described. However, as country of birth was not recorded in the dataset, a birth cohort approach could not be used to calculate the cumulative proportion of children entering care though other studies have found similar results when using both census denominator and birth cohort methods (Magruder & Shaw, 2008; Ubbesen et al., 2015). A further limitation is that, as the dataset did not contain detailed information related to care (e.g., support and interventions provided, parental contact, placement with siblings, etc.), our results provide only a crude description of the type of care children receive. Finally, the main limitation of this study is that the focus on first entries to OHC, and the following two years, means our analyses do not account for complex trajectories of care where multiple entries and exits throughout childhood are possible.

As the proportion of children entering OHC is a commonly used measure in social care research, our analyses can be used for cross-national comparisons. For example, the cumulative proportion of infants born in 2007 placed in OHC in England (0.6%) was similar to New Zealand and the US, higher than Australia or Sweden (0.3% each) and lower than Manitoba, Canada (2.9%) (Gilbert et al., 2009). The proportion of children born 1992–94 entering OHC by age 16 (2.9%) was similar to that reported in Denmark (3.4%): however, over time, rates of entry to OHC decreased in Denmark (Ubbesen et al., 2015) but increased in England. Exploration of differences and divergent trends such as these may be useful for informing policy development. Projections based on our results could also be used to plan future services. Since 1992 the proportion of children entering OHC has increased and, if patterns of entry to care observed for older cohorts of children continue, the overall proportion of children who enter OHC by age 18 will exceed 3.3% in the future. Such increases will have implications in terms of capacity and cost and need to be considered when developing children’s social welfare policies, planning services, and allocating resources. Our results also demonstrate that the proportion of children who are voluntarily placed in care has decreased over time. If this trend of increased need for legal intervention continues it will also have financial implications as the average cost of care proceedings to remove a children from their parents is £15,000 (Broadhurst & Mason, 2013). A further application of our analyses to policy makers is the evaluation of changes in practice with regard to social care policy. For example, in England there has been an increased focus on early intervention in recent years (Cabinet Office, 2011) and the increasing proportion of infants entering OHC evident in our analyses suggests a corresponding shift in practice over time. Permanence is also a central goal of the social care system and the decreasing proportion of children who experience more than one placement move indicates improvements have been made. However, these improvements were not evident in all age groups with no change over time among infants, despite the importance of stability of caregivers during this developmentally sensitive time (Berens & Nelson, 2015). In this way, our results can be used by social care practitioners to identify subgroups of children with potentially unmet needs.

Ethnic disproportionalities were evident among children in England with those of Black, Mixed, or Other ethnicity more likely to be placed in care. However, the scale of ethnic variation was not as pronounced as in other countries, such as the United States (Magruder & Shaw, 2008), and not all ethnic minorities were over-represented: as described elsewhere (Thoburn, Ashok, & Proctor, 2005) those of Asian ethnicity were significantly less likely to enter OHC. Our results also demonstrate that concurrent changes in the population’s ethnic composition appear to have had little impact on the overall proportion of children in care, and instead the greatest determinant was the small yet significant increase in the rate of entry to care among White children. Further work is required to understand the causes of this increase including the role of high profile child welfare cases, changes in social work practice, and increased diversity within the White population (e.g., due to immigration from Eastern European countries). Future research would also include exploration of trajectories of care among looked after children, beyond first entries and the following two years. For example, total time spent in OHC over childhood, mode of exit (e.g., adoption or return to parents), re-entry to OHC, and the relationship between these factors remains to be explored. It would also be useful to explore variation by local authority in entry to OHC, care characteristics and how these have changed over time, particularly in relation to differing social care budgets. Finally, an important next step would be to explore the effects of changes in OHC described in our results on outcomes in childhood and later life by linking social care data to health, education, and justice datasets. However, this would require collaboration across government departments and, in the absence of a common identifier, the development of effective algorithms for linkage.

Currently, one in thirty children in England experience at least one episode of OHC before their 18th birthday and, if current trends continue, this figure is set to increase in the future. The amount of time children spend in care also appears to be increasing in the short-term i.e. the two years following first entry. More children entering care and staying for longer will have financial implications for service providers and policy makers, particularly in the current context of economic austerity. OHC is an expensive intervention but the implications of the State assuming the caring role of the parent for a significant proportion of children need to be considered, not just in terms of economic costs but also in terms of the individual and societal well-being. For example, for some vulnerable children, levels of harm sufficient to justify entry to OHC care may potentially be prevented through early, intensive family support. For other children OHC is necessary to safeguard and improve their well-being and it is important that measures are taken to continually improve the services they receive. Our analyses highlight some changes over time in the characteristics of OHC that mean children are now more likely to be placed in a stable, family-type setting, which is likely to be beneficial in terms of a wide range of outcomes. Further research is required to understand the reasons for observed changes in practice and whether they are cost-effective, sustainable, and improve outcomes for children and society.

## Supplementary Material

Supplementary data associated with this article can be found, in the online version, at http://dx.doi.org/10.1016/j.chiabu.2015.10.020.

Supplementary Material

## Figures and Tables

**Table 1:** Characteristics of looked after children at first entry to out-of-home care, by year of birth (%).

	1992–94	1995–97	1998–00	2001–03	2004–06	2007/08	2009–11
**All children (*N*)**	19,751	17,415	13,058	11,001	10,070	6,141	7,328
*Gender*							
Male	54.5	52.2	53.4	52.7	52.0	52.7	51.6
Female	45.6	47.8	46.6	47.3	48.0	47.3	48.4

*Ethnicity*^a^							
White	72.6	75.4	77.9	76.4	75.3	75.4	78.4
Mixed	6.6	7.1	8.4	9.8	11.1	11.9	11.6
Asian	7.1	5.2	3.7	3.8	3.7	3.8	3.0
Black	9.3	9.2	8.0	8.5	8.4	6.8	5.3
Other	4.5	3.1	2.0	1.6	1.5	2.1	1.8
Unknown	27.4	19.0	5.8	0.2	0.5	0.6	1.8

**Infants (*N*)**	2,886	3,083	3,349	3,602	3,903	2,813	5,153
*Reason looked after*^b^							
Abuse or neglect	41.6	52.7	64.2	67.0	67.0	67.1	69.0
Child’s disability	0.9	1.4	1.0	0.9	0.8	0.6	0.4
Parental illness or disability	11.7	9.5	7.8	7.8	6.5	6.1	5.1
Family in acute stress	14.7	12.4	7.4	7.4	8.3	8.3	6.8
Family dysfunction	1.5	2.4	7.0	8.6	10.4	12.2	15.0
Socially unacceptable behaviour	0.6	0.8	0.4	0.2	0.4	0.2	0.4
Low income	0.0	0.1	0.2	0.2	0.2	0.2	0.2
Absent parenting	19.6	15.1	10.7	7.8	6.4	5.2	3.1
Other^c^	9.4	5.7	1.3	n/a	n/a	n/a	n/a

*Legal status*^d^							
Care order	14.2	19.9	26.1	29.5	29.9	29.5	35.0
Placement order	0.6	0.2	0.2	0.1	0.2	0.1	0.1
Child protection	14.6	17.8	19.6	18.2	17.0	13.6	12.1
Voluntary placements	70.6	62.0	54.1	52.3	52.9	56.8	52.8

**1–4 year olds (*N*)**	4,284	3,940	3,809	3,565	4,073	3,184	2,087
*Reason looked after*^b^							
Abuse or neglect	49.4	61.1	65.7	67.5	66.1	69.3	69.6
Child’s disability	1.9	1.9	1.3	1.0	0.8	0.4	0.6
Parental illness or disability	18.4	13.3	11.3	8.3	6.7	5.0	4.5
Family in acute stress	17.0	10.8	8.8	8.8	9.3	8.2	7.1
Family dysfunction	3.5	5.9	7.8	9.4	13.0	14.2	16.1
Socially unacceptable behaviour	0.8	0.6	0.4	0.5	0.4	0.6	0.9
Low income	0.1	0.3	0.3	0.5	0.3	0.2	0.3
Absent parenting	4.9	4.9	4.2	4.0	3.4	2.1	1.0
Other^c^	3.9	1.3	0.1	n/a	n/a	n/a	n/a

*Legal status*^d^							
Care order	14.0	20.7	24.8	25.2	23.2	25.7	28.3
Placement order	0.0	0.1	0.1	0.0	0.2	0.1	0.7
Child protection	16.6	19.5	18.7	22.5	23.2	22.7	20.9
Voluntary placements	69.3	59.7	56.4	52.2	53.3	51.5	50.1

**5–10 year olds (*N*)**	4,331	4,012	3,850	3,712	2,094	n/a	n/a
*Reason looked after*^b^							
Abuse or neglect	57.8	59.0	61.8	64.1	67.3		
Child’s disability	5.4	5.1	4.0	3.5	1.9		
Parental illness or disability	11.5	9.3	6.0	4.9	4.6		
Family in acute stress	10.2	9.4	9.9	8.7	8.9		
Family dysfunction	8.0	10.3	12.1	14.4	13.9		
Socially unacceptable behaviour	1.4	0.9	1.0	0.9	0.8		
Low income	0.3	0.3	0.3	0.2	0.4		
Absent parenting	4.9	5.8	4.9	3.2	2.1		
Other^c^	0.5	0.0	0.0	n/a	n/a		

*Legal status*^d^							
Care order	21.1	22.3	23.2	23.9	26.5		
Placement order	0.0	0.0	0.0	0.0	0.1		
Child protection	17.2	19.1	20.9	22.3	22.4		
Voluntary placements	61.6	58.6	55.8	53.8	51.0		

**11–15 year olds (*N*)**	6,052	5,461	2,050	n/a	n/a	n/a	n/a
*Reason looked after*^b^							
Abuse or neglect	34.5	42.4	55.4				
Child’s disability	4.7	4.5	4.8				
Parental illness or disability	2.7	3.0	3.1				
Family in acute stress	15.8	14.1	11.9				
Family dysfunction	18.5	20.3	17.8				
Socially unacceptable behaviour	9.6	6.6	3.1				
Low income	0.3	0.2	0.1				
Absent parenting	13.9	8.9	3.9				

*Legal status*^d^							
Care order	6.9	8.4	16.3				
Placement order	0.0	0.0	0.0				
Child protection	10.3	12.0	16.2				
Voluntary placements	77.9	76.0	66.2				
Youth justice	4.9	3.6	1.2				

**16+ year olds (*N*)**	2,198	919	n/a	n/a	n/a	n/a	n/a
*Reason looked after*^b^							
Abuse or neglect	17.3	22.6					
Child’s disability	4.3	4.8					
Parental illness or disability	0.9	0.5					
Family in acute stress	13.2	14.5					
Family dysfunction	20.6	21.7					
Socially unacceptable behaviour	6.4	8.5					
Low income	1.5	0.5					
Absent parenting	35.9	26.9					

*Legal status*^d^							
Care order	1.3	0.9					
Placement order	0.0	0.0					
Child protection	2.6	4.5					
Voluntary placements	91.8	86.8					
Youth justice	4.3	7.8					

a Calculated as % of those with known ethnicity.

b Only one reason looked after can be selected for each child; if more than one is applicable then the highest ordered reason should be selected.

c “Reason looked after” replaced the more detailed “category of need” variable in 2000. Categories of need with no comparable reason looked after have been classed here as “Other”. Codes in each reason looked after category are described in Supplementary Table 1.

d Care order = full or interim care order; placement order = freeing or placement order; child protection = under child assessment order, police protection or emergency protection order; voluntary = single period of accommodation under Section 20 of the Children Act 1989; youth justice = detained under PACE (Police and Criminal Evidence Act), sentenced to CYPA 1969 supervision order with residence requirement, on remand or committed for trial or sentence. Codes in each legal status category are described in Supplementary Table 2.

e A small number of children born in 2007/08 and aged 5 at first entry to care (*n* = 144) or born 2001/03 and aged 11 (*n* = 122) have been excluded from this table.

**Table 2:** Cumulative percentage of children entering OHC by year of birth, age, gender and ethnicity.

	Year of birth	By age (years)						
		1	4	6	9	12	15	18
All	1992–94	0.46	1.01	1.27	1.61	1.98	2.57	3.25
	1995–97	0.51	1.04	1.28	1.61	1.91	2.48	
	1998–00	0.58	1.04	1.27	1.55	1.87		
	2001–03	0.65	1.16	1.41	1.80			
	2004–06	0.64	1.17	1.47				
	2007/08	0.64	1.25					
	2009–11	0.81						

Boys	1992–94	0.49	1.06	1.32	1.69	2.11	2.71	3.46
	1995–97	0.54	1.07	1.33	1.68	2.00	2.54	
	1998–00	0.60	1.09	1.32	1.62	1.97		
	2001–03	0.67	1.20	1.46	1.97			
	2004–06	0.64	1.19	1.49				
	2007/08	0.66	1.29					
	2009–11	0.81						

Girls	1992–94	0.41	0.95	1.20	1.52	1.85	2.43	3.03
	1995–97	0.47	0.99	1.22	1.52	1.80	2.40	
	1998–00	0.53	0.99	1.20	1.47	1.76		
	2001–03	0.60	1.11	1.36	1.73			
	2004–06	0.61	1.13	1.44				
	2007/08	0.59	1.19					
	2009–11	0.76						

White	2001–03	0.59	1.07	1.30	1.64			
	2004–06	0.59	1.07	1.34				
	2007/08	0.59	1.15					
	2009–11	0.77						

Mixed	2001–03	1.87	3.03	3.55	4.22			
	2004–06	1.89	2.96	3.60				
	2007/08	1.63	2.97					
	2009–11	1.80						

Asian	2001–03	0.28	0.49	0.61	0.84			
	2004–06	0.23	0.44	0.63				
	2007/08	0.27	0.52					
	2009–11	0.23						

Black	2001–03	1.16	2.53	3.24	4.52			
	2004–06	1.21	2.66	3.51				
	2007/08	1.16	2.37					
	2009–11	1.10						

Other	2001–03	1.08	1.92	2.21	2.74			
	2004–06	0.81	1.54	1.99				
	2007/08	0.96	1.83					
	2009–11	0.85						

Ethnicity was poorly recorded for children born between 1992 and 2000 (6–27% missing) therefore ethnic-specific rates could not be calculated for the earlier year of birth groups. ETHPOP data (Wohland et al., 2015) was used as the source of denominator data by ethnicity.

**Table 3:** Contribution of changes in population weights and ethnic-specific rates of entry to care to overall changes in the proportion of children entering out-of-home care (absolute percentage points).

	By age 1	By age 2	By age 3	By age 4	By age 5	By age 6	By age 7
Actual overall change	0.14	0.15	0.04	0.08	0.08	0.06	0.05

Attributable to change in population weight							
- White	–0.03	–0.03	–0.03	–0.03	–0.03	–0.02	–0.02
- Mixed	0.02	0.02	0.02	0.02	0.02	0.02	0.01
- Asian	0.005	0.01	0.01	0.01	0.01	0.005	0.005
- Black	0.005	0.01	0.01	0.01	0.01	0.004	0.004
- Other	0.01	0.01	0.01	0.01	0.01	0.005	0.005

Attributable to change in rate of entry to OHC							
- White	0.15	0.15	0.04	0.07	0.07	0.04	0.03
- Mixed	–0.003	–0.01	–0.01	–0.003	–0.001	0.002	0.004
- Asian	–0.004	–0.005	–0.002	0.003	0.003	0.002	0.001
- Black	–0.002	–0.01	–0.004	–0.005	–0.01	0.01	0.005
- Other	–0.003	–0.003	–0.001	–0.001	0.001	–0.002	–0.004

Calculated change^a^	0.15	0.14	0.04	0.08	0.08	0.07	0.04

OHC = out-of-home care. Additional details related to decomposition methodology and results given in Supplementary Box 1.

a As ethnicity was not recorded for all individuals there may be slight differences between actual overall change and the change calculated by summing ethnic-specific changes.

**Table 4:** Characteristics of out-of-home care during the two years following first entry, by age and year of birth.


Age group at first entry		1992				2000				2008		
		Family	Group	Other		Family	Group	Other		Family	Group	Other

(i) First placement type^a^										
<1 year		90.1%	8.4%	1.6%		89.9%	9.9%	0.3%		91.2%	8.5%	0.3%
<1–4 years		95.9%	2.3%	1.8%		98.1%	1.8%	0.2%		99.2%	0.5%	0.4%
<5–10 years		90.7%	8.0%	1.3%		95.8%	3.5%	0.6%				
<11–15 years^b^		73.4%	23.0%	3.5%								
<16+ years^b^		30.0%	34.6%	35.3%								

Age group at first entry		1992				2000				2008		
		Mean	Range	>1 change		Mean	Range	>1 change		Mean	Range	>1 change

(ii) Placement changes^c^												
<1 year		1.59	0–51	32.2%		1.49	0–10	37.6%		1.25	0–9	31.0%
<1–4 years		1.95	0–67	38.4%		1.26	0–30	32.2%		1.08	0–16	13.9%
<5–10 years		1.70	0–60	35.9%		1.01	0–18	22.9%				
<11–15 years		2.03	0–135	39.2%								

Age group at first entry			1992				2000				2008	
			Mean	Median			Mean	Median			Mean	Median

(iii) Time in care (weeks)^d^												
<1 year			49	39			66	75			70	80
<1–4 years			45	24			63	78			67	83
<5–10 years			65	94			67	95				
<11–15 years			57	61								


a Family placement = placed for adoption and kin or stranger foster care; group placement = children’s home, health-related residential setting, residential school or other supported accommodation; other placement = independent living, in custody or other placement. Codes in each placement category are described in Supplementary Table 3.

b Of children born in 1992, 1% of those entering care aged 11–15 and 33% of those aged 16+ were placed in independent living.

c Range and mean number of placement changes in the two years following first entry to out-of-home care and the proportion of children who experienced more than one placement change during this time.

d Mean and median weeks in care in the two years following first entry to out-of-home care.
