# Supplementary material for: Changes in first entry to out-of-home care from 1992 to 2012 among children in England
Source: Child Abuse Negl. Author manuscript; Available in PMC 2018 Oct 29. (PMC6205623; doi:10.1016/j.chiabu.2015.10.020)
Supplement: Supplementary Material [file NIHMS80111-supplement-Supplementary_Material.docx]

**Supplementary Material**

Supplementary Figure 1

*Creation of study datasets*

*Contained details of all episodes of care from January 1, 1992, to December 31, 2012, for looked-after children born between January 1, 1992, and December 31, 2011, with complete care histories. This sample represents one-third of all looked after children.*

**Original dataset**

92,190 child IDs

405,696 episodes

Exclude individuals:

- Who have no OHC recorded i.e. are placed only with parents (n=2,243)
- Whose placement location is unknown (n=24)
- Who are in OHC for respite reasons only (n=5,249)

*Characteristics described in Table 1. This sample was used to calculate the cumulative proportion of children entering OHC for non-respite reasons during their childhood (Fig. 1, Table 2) and explore the effect of changing ethnic distribution among children in England on the proportion entering OHC (Table 3).*

**Out-of-home care dataset**

84,674 child IDs

355,836 episodes

Exclude individuals:

- Not born in 1992, 2000 or 2008 (n=70,974)

**Born 1992, 2000 & 2008**

13,700 child IDs

60,471 episodes

*Contained details of all episodes of out-of-home care for non-respite reasons from January 1, 1992, to December 31, 2012for children born in 1992, 2000 or 2008 (N= 6,649, 4,033 and 3,018 respectively). This sample was used to explore changes over time in first placement type (Table 4(i)).*

Exclude individuals without two years follow-up because they entered OHC:

- In 2011/12 (n=1,275)
- Aged 16/17 (n=671)

*Contained details of looked-after children born in 1992, 2000 or 2008 with at least two years of follow-up. This sample was used to describe duration of care and placement stability in the two years following first entry to OHC (Table 4 (ii) & (iii)).*

**Two year follow up**

11,754 child IDs

37,303 episodes

Supplementary box 1

*Exploring the effects of changing ethnic distribution on rates of entry to OHC*

**Is the overall increase in the cumulative proportion of children entering out-of-home care due to changes in the ethnic composition of the child population?**

**Background**

Over time the proportion of children entering out-of-home care (OHC) appears to have increased, for example, from 0.65% of infants born 2001-03 to 0.81% of those born 2009-11. This rate of entry to OHC varies by ethnicity (with lower rates observed among White and Asian children) and has generally decreased among ethnic minorities and increased among White children over time (Table 1). The ethnic composition of the child population has also changed over the same time period, for example the proportion of ethnic minority infants has increased from 15.8% of those born 2001-2003 to 19.6% of those born 2009-11 (see Table 1).

Table 1: *Ethnic composition and rates of entry to out-of-home care for infants by year of birth*

|  | 2001-03 | | 2009-11 | | Change | | Average | |
| --- | --- | --- | --- | --- | --- | --- | --- | --- |
|  | Population  % | Rate of entry | Population  % | Rate of entry | Population  % | Rate of entry | Population  % | Rate of entry |
| White | 84.2 | 0.60 | 80.4 | 0.77 | -3.8 | 0.17 | 82.3 | 0.69 |
| Mixed | 4.3 | 1.86 | 5.2 | 1.80 | 0.9 | -0.06 | 4.8 | 1.83 |
| Asian | 7.4 | 0.28 | 9.2 | 0.23 | 1.8 | -0.05 | 8.3 | 0.26 |
| Black | 3.3 | 1.16 | 3.7 | 1.10 | 0.4 | -0.05 | 3.5 | 1.13 |
| Other | 0.9 | 1.09 | 1.5 | 0.84 | 0.7 | -0.25 | 1.2 | 0.97 |

The cumulative proportion of children in OHC at any time is simply a weighted sum of those in care by ethnicity at any time *t*. It can be calculated by summing each ethnic-specific rate of entry times its weight (i.e. the proportion of the child population of that ethnicity). For example the overall cumulative proportion of infants entering OHC in 2001-03 was 0.65% which can be calculated using values from Table 1 (Fig. 1). To explore changes over time the equation can be differentiated with respect to time (*t*) using average weights for the two time periods of interest. Using rules of calculus this gives the equation used in this study as described in Figure 2.

Figure 1: *Calculation of overall change among infants using ethnic-specific rates and population weights*

Cumulative proportion ≈ (W_white_)(R_white_) + (W_mixed_)(R_mixed_) + (W_Asian_)(R_Asian_) + (W_black_)(R_black_) + (W_other_)(R_other_)

where W = ethnic-specific weight (i.e. proportion of population of specified ethnicity)

R = ethnic-specific rate of entry (i.e. proportion of specified ethnicity that enter OHC)

Cumulative proportion ≈ (0.84)(0.60) + (0.04)(1.86) + (0.07)(0.28) + (0.03)(1.16) + (0.01)(1.09)

≈ (0.50) + (0.08) + (0.02) + (0.04) + (0.01)

≈ 0.65

**Methods**

First, the change in the ethnic-specific weights and rates among infants between 2001-03 and 2009-11 and the average weight and rate for each ethnic group were calculated (Table 1). To explore the relative contribution of (1) the changing ethnic composition of the infant population and (2) the changing ethnic-specific rates of entry to the overall increase in the cumulative proportion entering OHC these figures were then substituted into a mid-point estimate decomposition formula (Fig. 2).The same approach was then used to decompose the change in the cumulative proportion of children entering care up to age 6 (the oldest age for which there are two or more comparable year of birth groups) into components attributable to change in ethnic-specific rates and weights during that time.

Figure 2: *Decomposition of overall change into group components using midpoint estimates*

∆Cumulative proportion ≈ ($\bar{W}$_w_)(∆R_w_) + ($\overline{W}$_m_)(∆R_m_) + ($\overline{W}$_a_)(∆R_a_) + ($\overline{W}$_b_)(∆R_b_) + ($\overline{W}$_o_)(∆R_o_)

+ ($\bar{R}$_w_)(∆W_w_) + ($\overline{R}$_m_)(∆W_m_) + ($\overline{R}$_a_)(∆W_a_) + ($\overline{R}$_b_)(∆W_b_) + ($\overline{R}$_o_)(∆W_o_)

where $\overline{W}$ = average ethnic-specific weight (i.e. proportion of population of specified ethnicity)

∆W = change in ethnic-specific weight

$\overline{R}$ = average ethnic-specific rate of entry (i.e. proportion of ethnic group that enter OHC)

∆R = change in ethnic-specific rate of entry to care

**Results**

From the decomposition equation (Fig. 3) we can see that, for infants, changes in weights (i.e. the ethnic composition of the infant population) has had a minimal effect on the overall proportion entering care. For example, the 3.8% decrease in the proportion of white infants accounted for an absolute decrease of 0.03% in the overall proportion of children entering care while the 1.8% increase in Asian children accounted for an absolute increase of <0.1%. The increase in the cumulative proportion of children entering care can instead be attributed to changes in the ethnic-specific rates of entry to care. Specifically, the increase in the rate of entry among white children from 0.60% to 0.77% accounted for an absolute increase of 0.14% in the overall proportion of children entering care. The reduction in rates of entry among all other ethnic minority groups accounted for a negligible decrease of just 0.01% in the overall proportion.

Figure 3: *Decomposition of overall change among infants into group components using midpoint estimates*

∆Cumulative proportion ≈ (0.823)(0.17) + (0.048)(-0.06) + (0.083)(-0.05) + (0.035)(-0.05) + (0.012)(-0.25)

+ (0.69)(-0.038) + (1.83)(0.009) + (0.26)(0.018) + (1.13)(0.004) + (0.97)(0.007)

≈ (0.14) + (-0.003) + (-0.004) + (-0.002) + (-0.003)

+ *(-0.03*) + *(0.02)* + *(0.005)* + *(0.005)* + *(0.007)*

≈ 0.14

Similar results were observed for all other ages with changes in the overall cumulative proportion of children entering OHC over time attributable primarily to an increase in the rate of entry to care among white children and the changing ethnic composition of the child population making little contribution (Table 2).

Table 2: *Decomposition of overall change in cumulative proportion into components attributable to changes in ethnic-specific population weights and rates of entry to care for infants to 6 year olds*

| Change attributable to changes in ethnic-specific weights (percentage points) | | | | | | | |
| --- | --- | --- | --- | --- | --- | --- | --- |
|  | Infants | 1 year | 2 years | 3 years | 4 years | 5 years | 6 years |
| White | -0.03 | -0.03 | -0.03 | -0.03 | -0.03 | -0.02 | -0.02 |
| Mixed | 0.02 | 0.02 | 0.02 | 0.02 | 0.02 | 0.02 | 0.01 |
| Asian | 0.01 | 0.01 | 0.01 | 0.01 | 0.01 | 0.01 | 0.01 |
| Black | 0.01 | 0.01 | 0.01 | 0.01 | 0.01 | 0.004 | 0.004 |
| Other | 0.01 | 0.01 | 0.01 | 0.01 | 0.01 | 0.01 | 0.01 |
| Change attributable to changes in ethnic-specific rates (percentage points) | | | | | | | |
|  | Infants | Age 1 | Age 2 | Age 3 | Age 4 | Age 5 | Age 6 |
| White | **0.15** | **0.15** | **0.04** | **0.07** | **0.07** | **0.04** | **0.03** |
| Mixed | -0.003 | -0.01 | -0.01 | -0.003 | -0.001 | 0.002 | 0.004 |
| Asian | -0.004 | -0.01 | -0.002 | 0.003 | 0.003 | 0.002 | 0.001 |
| Black | -0.002 | -0.01 | -0.004 | -0.01 | -0.01 | 0.01 | 0.01 |
| Other | -0.003 | -0.003 | -0.001 | -0.001 | 0.001 | -0.002 | -0.004 |
| Overall calculated change in cumulative proportion (percentage points) | | | | | | | |
|  | Infants | Age 1 | Age 2 | Age 3 | Age 4 | Age 5 | Age 6 |
| All | 0.14 | 0.15 | 0.04 | 0.08 | 0.08 | 0.06 | 0.04 |

Greatest contributor to overall change highlighted in bold.

**Conclusion**

The increase over time in the cumulative proportion of children entering OHC up to age 7 can be attributed primarily to the increase in the rate of entry to care among white children and not to the changing ethnic composition of the child population.

Supplementary Table 1

*Codes included in reason looked after categories*

| **Category of need** | **Category of need code** | **Description** (27) | **Reason looked after code** | **Description** |
| --- | --- | --- | --- | --- |
| Abuse or neglect | N1 | Children in need as a result of, or at risk of, abuse or neglect. | 10 | Preventative child welfare |
|  |  |  | 20 | Abuse or neglect |
| Child’s disability | N2 | Children and their families whose main need for services arises out of the child’s disability, illness or intrinsic condition. | 14 | Child has learning disability |
|  |  |  | 15 | Child has physical/sensory disability |
|  |  |  | 16 | Child has both physical/sensory and learning disability |
| Parental illness or disability | N3 | Children whose main need for services arises because of the capacity of their parents to care for them is impaired by disability, illness, mental illness, or addictions | 1 | Ill-health of parent(s) |
| Family in acute stress | N4 | Children whose needs arise from living in a family going through temporary crisis such that parenting capacity is diminished and some of the children’s needs are not being adequately met. | 4 | Family is homeless |
|  |  |  | 7 | Parent(s) need relief |
| Family dysfunction | N5 | Children whose needs arise mainly out of their living with families where the parenting capacity is chronically inadequate. | - |  |
| Socially unacceptable behaviour | N6 | Children and families whose need for services arise primarily out of their children’s behaviour impacting detrimentally on the community. | 21 | Risky behaviour |
|  |  |  | 22 | Child has been found guilty of an offence |
|  |  |  | 23 | Child is accused of an offence |
| Low income | N7 | Children, either living in families or independently, whose need for services arises mainly from being independent on an income below the standard state entitlements. | - |  |
| Absent parenting | N8 | Children whose need for services arises mainly from having no parents available to provide for them. Children whose parents decide it is in the best interests of the child to be adopted would be included in this category. | 2 | No parent or guardian |
|  |  |  | 3 | Abandoned or lost |
|  |  |  | 5 | Parent(s) in prison |
|  |  |  | 9 | Child aged 16+ is homeless |
|  |  |  | 11 | Adoption at request of parent(s) |
| Other* | - | Reason looked after codes that have no equivalent category of need | 8 | Child requested to be looked after |
|  |  |  | 12 | Child freed for adoption |
|  |  |  | 13 | Breakdown of adoptive family |
|  |  |  | **6, 8, 19,** 29 | Other reason (not specified) |

Supplementary Table 1 shows the reason looked after codes (used pre-2000) associated with each category of need code. *”Other” category was created in this study to include reasons looked after without an equivalent category of need code.

Supplementary Table 2

*Codes included in legal status categories*

| **Category** | **Legal status** | **Code** | **Description** | **In use** |
| --- | --- | --- | --- | --- |
| Care order | Interim care order | C1 | Local authority granted legal responsibility for the child for up to 28 days (must be renewed) | 1992- |
|  | Full care order | C2 | Local authority granted legal responsibility for the child (not time limited) | 1992- |
|  | Other care order | C9 | Local authority granted legal responsibility for the child | 1992-2000 |
| Placement order | Freeing order | D1 | Freeing order granted, child is freed for adoption | 1992-2005 |
|  | Placement order | E1 | Placement order granted, child is freed for adoption | 2006- |
| Child protection | Police protection | L1 | Child is under police protection and in Local Authority accommodation | 1992- |
|  | Emergency protection order | L2 | Child subject to emergency protection order as there are reasonable grounds for believing there is immediate risk of significant harm | 1992- |
|  | Child assessment order | L3 | Child subject to child assessment order as there are suspicions, but no firm evidence, of actual or likely significant harm | 1992- |
| Youth justice | On remand | J1 | Child is on remand, or committed for trial or sentence, and accommodated by Local Authority | 1992- |
|  | PACE detainment | J2 | Child is helping police with their enquiries and detained in Local Authority accommodation under Police and Criminal Evidence Act 1984 | 1992- |
|  | Supervision order | J3 | Child is placed under the supervision of the Local Authority and they must provide accommodation | 1992- |
| Voluntary* | Single section 20 accommodation | V2 | Single period of accommodation under Section 20 of the Children Act 1989 because the child is lost or abandoned, no person has parental responsibility for them or the person caring for them cannot provide suitable accommodation or care | 1992- |

Supplementary Table 2 shows the current and historic legal status codes grouped together in this study to create legal status categories and the years during which they were in use. *Children looked-after for respite reasons (not included in this analysis) were recorded using the voluntary legal status codes V1, V3 and V4 which indicate a child was accommodated “under an agreed series of short-term breaks”.

Supplementary Table 3

*Codes included in placement categories*

| **Category** | **Placement** | **Code** | **Description** | **In use** |
| --- | --- | --- | --- | --- |
| Family care | Placed for adoption | A1 | Placed for adoption with parental /guardian consent (or under freeing order) not with current foster carer | 1992-2006 |
|  |  | A2 | Placed for adoption with parental /guardian consent (or under freeing order) with current foster carer | 1992-2006 |
|  |  | A3 | Placed for adoption with parental /guardian consent (or under freeing order) with current foster carer | 2007- |
|  |  | A4 | Placed for adoption with parental /guardian consent (or under freeing order) not with current foster carer | 2007- |
|  |  | A5 | Placed for adoption with placement order with current foster carer or with freeing order where parental/guardian consent was dispensed by courts | 2007- |
|  |  | A6 | Placed for adoption with placement order not with current foster carer or with freeing order where parental/guardian consent was dispensed by courts | 2007- |
|  | Foster care (kin) | F9 | Foster placement with relative or friend | 1992-2000 |
|  |  | F1 | Foster placement with relative or friend inside Local Authority boundary | 2001-2008 |
|  |  | F4 | Foster placement with relative or friend outside Local Authority boundary | 2001-2008 |
|  |  | Q1 | Foster placement with relative or friend | 2009- |
|  | Foster care (stranger) | F8 | Foster placement with other foster carer | 1992-2000 |
|  |  | F2 | Foster placement with other foster carer inside Local Authority boundary, provided by Local Authority | 2001-2008 |
|  |  | F3 | Foster placement with other foster carer inside Local Authority boundary, arranged through agency | 2001-2008 |
|  |  | F5 | Foster placement with other foster carer outside Local Authority boundary, provided by Local Authority | 2001-2008 |
|  |  | F6 | Foster placement with other foster carer outside Local Authority boundary, arranged through agency | 2001-2008 |
|  |  | Q2 | Foster placement with other foster carer | 2009- |
| Group care | Children’s home | H3 | Children’s homes inside Local Authority boundary | 1992-2008 |
|  |  | H4 | Children’s homes outside Local Authority boundary | 1992-2008 |
|  |  | K2 | Children’s homes | 2009- |
|  | Residential care home | R1 | Residential care home | 1992- |
|  |  | R2 | NHS/Health Trust or other establishment providing medical or nursing care | 1992- |
|  | Residential school | S1 | All residential schools, except where dual-registered as a school and children’s home | 1997- |
|  | Other residential accommodation | H9 | Residential accommodation not subject to Children’s homes regulations but where formal support or supervision is provided | 1992-2000 |
|  |  | H5 | Residential accommodation not subject to Children’s homes regulations but where formal support or supervision is provided | 2001- |
|  |  | R3 | Family centre or mother and baby unit | 1992- |
| Other | Independent living | P2 | Independent living e.g. in flat, lodgings, bedsit, B&B or with friends, with or without formal support | 2006- |
|  |  | P3 | Residential employment including employment training and apprenticeships where accommodation is provided | 2001- |
|  | Secure unit | H1 | Secure unit inside Local Authority boundary | 1992-2005 |
|  |  | H2 | Secure unit outside Local Authority boundary | 1992-2005 |
|  |  | K1 | Secure unit | 2006- |
|  | Absent from placement | M1 | Absent from agreed placement but whereabouts known to social services (in refuge) | 1992- |
|  |  | M2 | Absent from agreed placement but whereabouts known to social services (not in refuge) | 1992- |
|  |  | M3 | Whereabouts unknown | 1992- |
|  | Other | R4 | Glenthorne Youth Treatment Centre | 1992-2003 |
|  |  | R5 | Young Offender Institute or prison | 1992- |
|  |  | Z1 | Other placements | 1992- |

Supplementary Table 3 shows the current and historic placement codes grouped together in this study to create placement categories and the years during which they were in use. Placements with parents (not included in this analysis) were recorded using code P1 which indicates a child was “placed with own parents of person with parental responsibility.”
